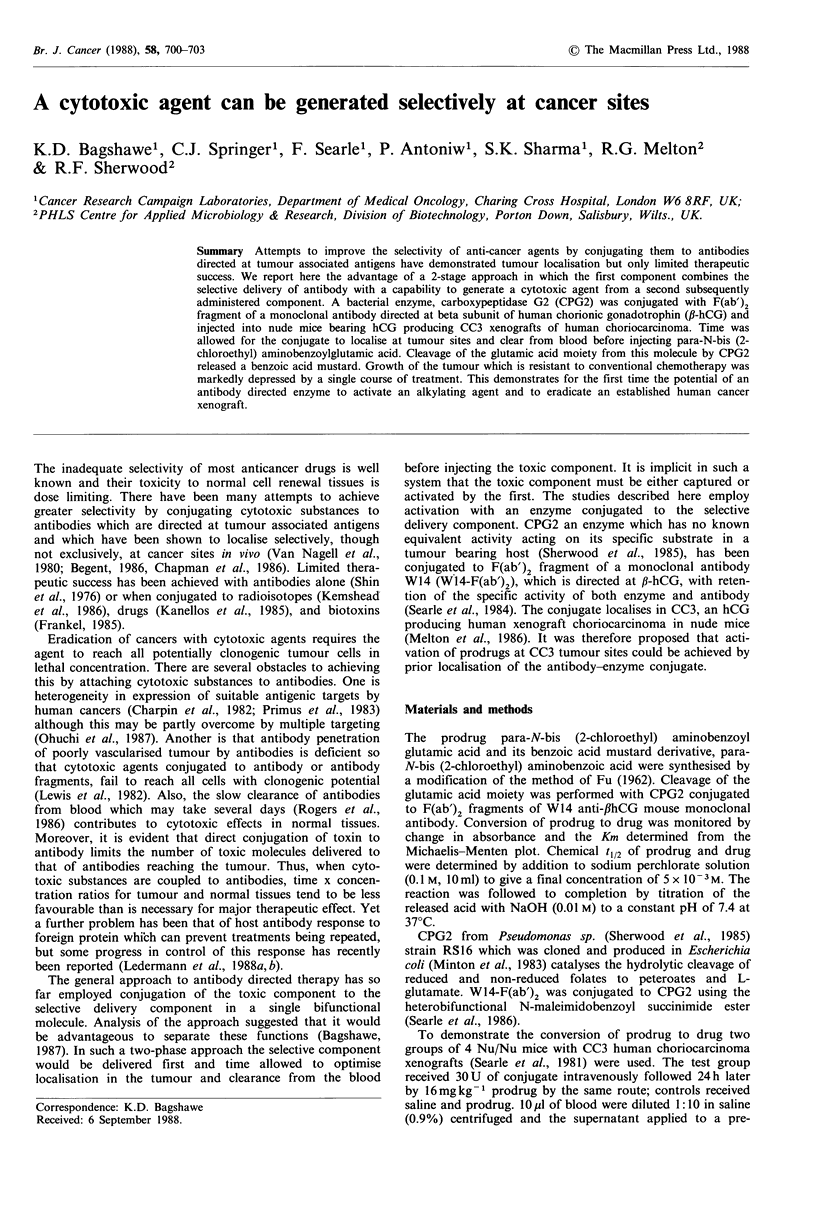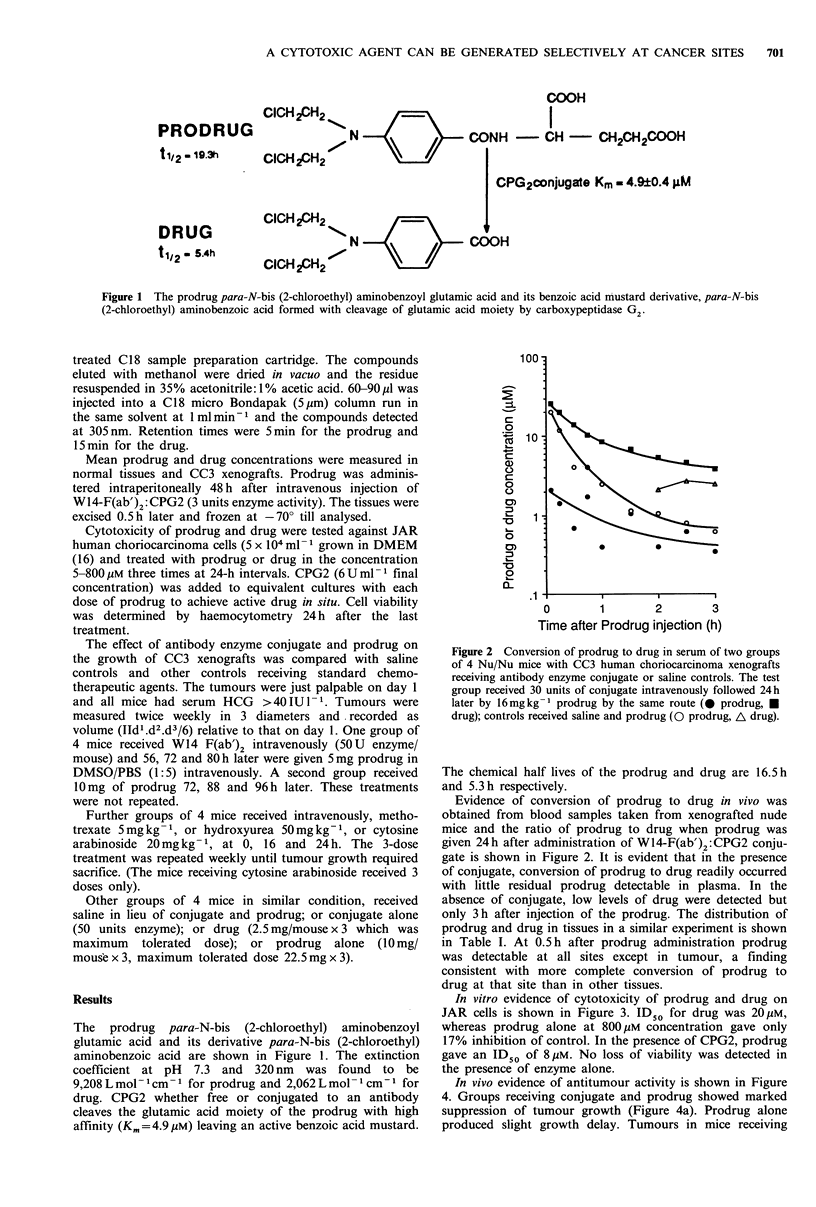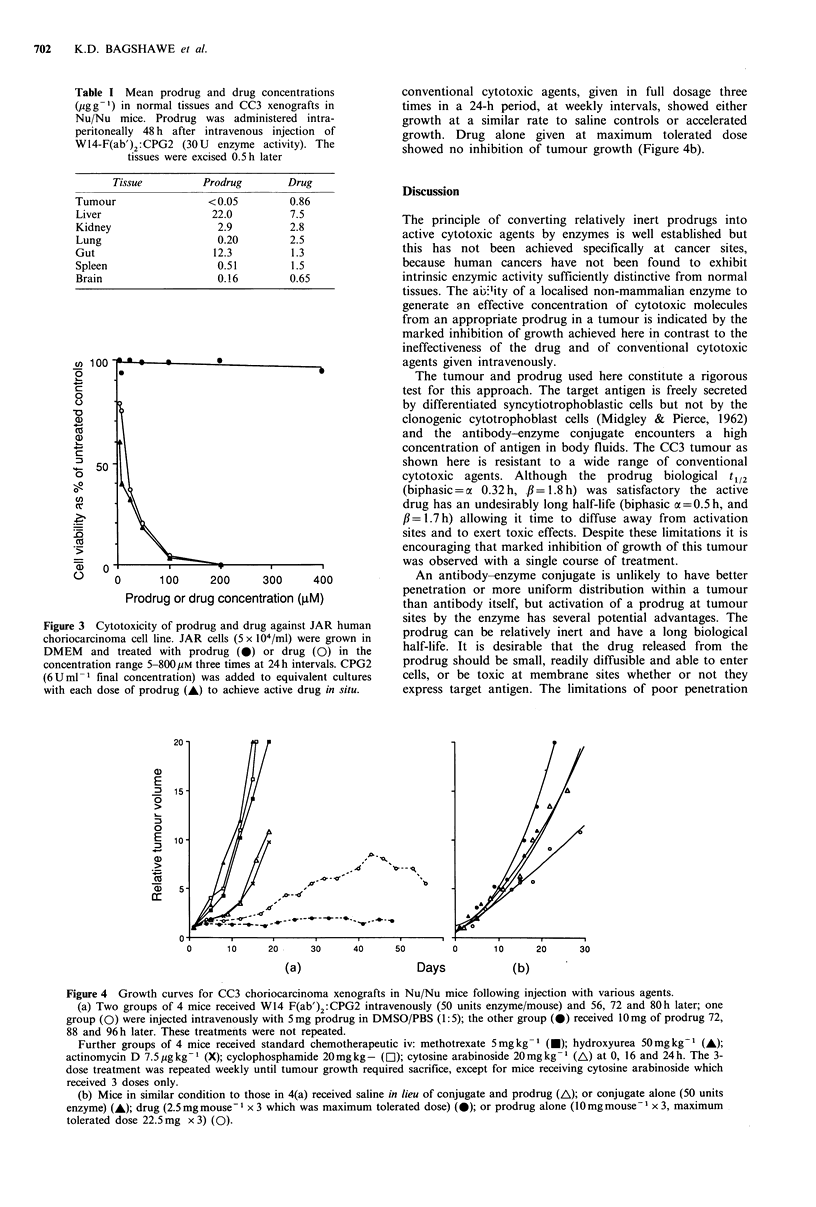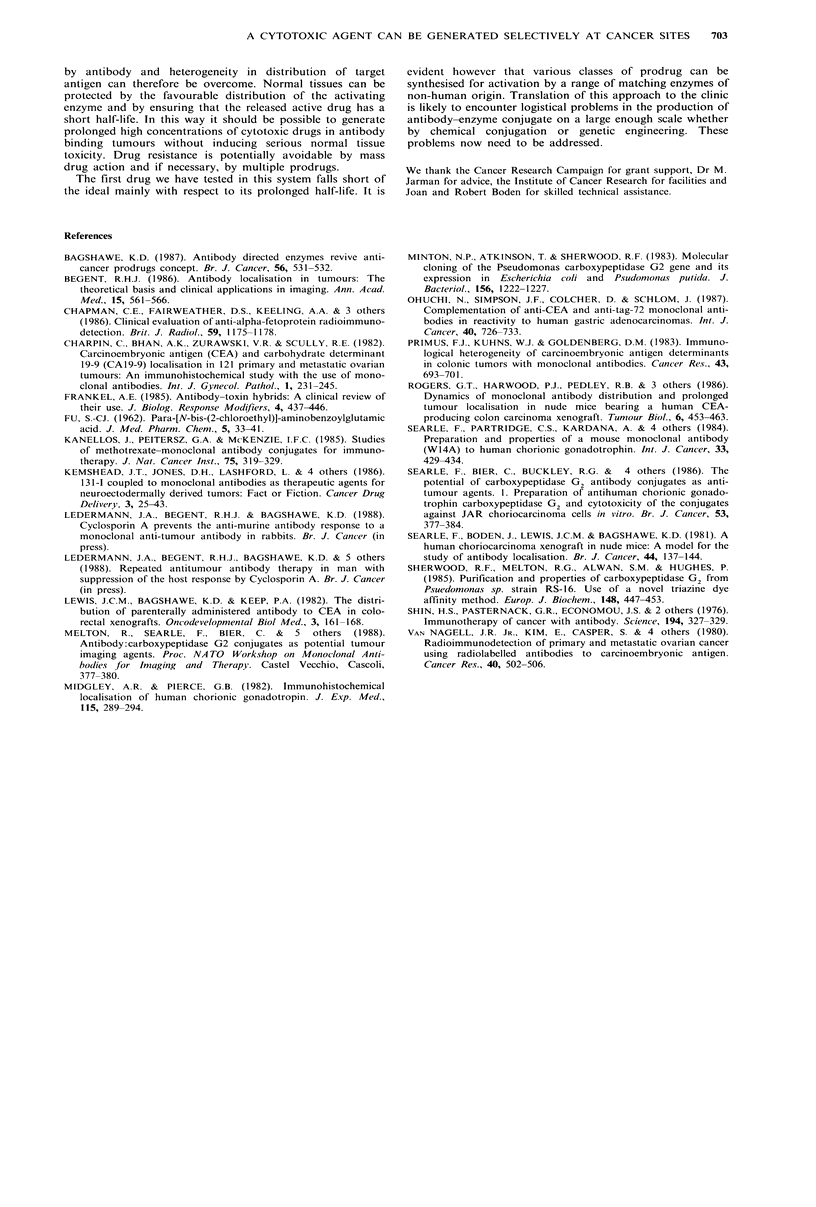# A cytotoxic agent can be generated selectively at cancer sites.

**DOI:** 10.1038/bjc.1988.293

**Published:** 1988-12

**Authors:** K. D. Bagshawe, C. J. Springer, F. Searle, P. Antoniw, S. K. Sharma, R. G. Melton, R. F. Sherwood

**Affiliations:** Department of Medical Oncology, Charing Cross Hospital, London, UK.

## Abstract

Attempts to improve the selectivity of anti-cancer agents by conjugating them to antibodies directed at tumour associated antigens have demonstrated tumour localisation but only limited therapeutic success. We report here the advantage of a 2-stage approach in which the first component combines the selective delivery of antibody with a capability to generate a cytotoxic agent from a second subsequently administered component. A bacterial enzyme, carboxypeptidase G2 (CPG2) was conjugated with F(ab')2 fragment of a monoclonal antibody directed at beta subunit of human chorionic gonadotrophin (beta-hCG) and injected into nude mice bearing hCG producing CC3 xenografts of human choriocarcinoma. Time was allowed for the conjugate to localise at tumour sites and clear from blood before injecting para-N-bis (2-chloroethyl) aminobenzoylglutamic acid. Cleavage of the glutamic acid moiety from this molecule by CPG2 released a benzoic acid mustard. Growth of the tumour which is resistant to conventional chemotherapy was markedly depressed by a single course of treatment. This demonstrates for the first time the potential of an antibody directed enzyme to activate an alkylating agent and to eradicate an established human cancer xenograft.


*Br. J. Cancer* (1988), **58**, 700–703
© The Macmillan Press Ltd., 1988

### A cytotoxic agent can be generated selectively at cancer sites

K.D. Bagshawe[1], C.J. Springer[1], F. Searle[1], P. Antoniw[1], S.K. Sharma[1], R.G. Melton[2] & R.F. Sherwood[2]

[1]*Cancer Research Campaign Laboratories, Department of Medical Oncology, Charing Cross Hospital, London W6 8RF, UK;* [2]*PHLS Centre for Applied Microbiology & Research, Division of Biotechnology, Porton Down, Salisbury, Wilts., UK.*

**Summary** Attempts to improve the selectivity of anti-cancer agents by conjugating them to antibodies directed at tumour associated antigens have demonstrated tumour localisation but only limited therapeutic success. We report here the advantage of a 2-stage approach in which the first component combines the selective delivery of antibody with a capability to generate a cytotoxic agent from a second subsequently administered component. A bacterial enzyme, carboxypeptidase G2 (CPG2) was conjugated with $F(ab')_2$ fragment of a monoclonal antibody directed at beta subunit of human chorionic gonadotrophin (β-hCG) and injected into nude mice bearing hCG producing CC3 xenografts of human choriocarcinoma. Time was allowed for the conjugate to localise at tumour sites and clear from blood before injecting para-N-bis (2-chloroethyl) aminobenzoylglutamic acid. Cleavage of the glutamic acid moiety from this molecule by CPG2 released a benzoic acid mustard. Growth of the tumour which is resistant to conventional chemotherapy was markedly depressed by a single course of treatment. This demonstrates for the first time the potential of an antibody directed enzyme to activate an alkylating agent and to eradicate an established human cancer xenograft.

The inadequate selectivity of most anticancer drugs is well known and their toxicity to normal cell renewal tissues is dose limiting. There have been many attempts to achieve greater selectivity by conjugating cytotoxic substances to antibodies which are directed at tumour associated antigens and which have been shown to localise selectively, though not exclusively, at cancer sites *in vivo* (Van Nagell *et al.*, 1980; Begent, 1986, Chapman *et al.*, 1986). Limited therapeutic success has been achieved with antibodies alone (Shin *et al.*, 1976) or when conjugated to radioisotopes (Kemshead *et al.*, 1986), drugs (Kanellos *et al.*, 1985), and biotoxins (Frankel, 1985).

Eradication of cancers with cytotoxic agents requires the agent to reach all potentially clonogenic tumour cells in lethal concentration. There are several obstacles to achieving this by attaching cytotoxic substances to antibodies. One is heterogeneity in expression of suitable antigenic targets by human cancers (Charpin *et al.*, 1982; Primus *et al.*, 1983) although this may be partly overcome by multiple targeting (Ohuchi *et al.*, 1987). Another is that antibody penetration of poorly vascularised tumour by antibodies is deficient so that cytotoxic agents conjugated to antibody or antibody fragments, fail to reach all cells with clonogenic potential (Lewis *et al.*, 1982). Also, the slow clearance of antibodies from blood which may take several days (Rogers *et al.*, 1986) contributes to cytotoxic effects in normal tissues. Moreover, it is evident that direct conjugation of toxin to antibody limits the number of toxic molecules delivered to that of antibodies reaching the tumour. Thus, when cytotoxic substances are coupled to antibodies, time x concentration ratios for tumour and normal tissues tend to be less favourable than is necessary for major therapeutic effect. Yet a further problem has been that of host antibody response to foreign protein which can prevent treatments being repeated, but some progress in control of this response has recently been reported (Ledermann *et al.*, 1988*a*,*b*).

The general approach to antibody directed therapy has so far employed conjugation of the toxic component to the selective delivery component in a single bifunctional molecule. Analysis of the approach suggested that it would be advantageous to separate these functions (Bagshawe, 1987). In such a two-phase approach the selective component would be delivered first and time allowed to optimise localisation in the tumour and clearance from the blood before injecting the toxic component. It is implicit in such a system that the toxic component must be either captured or activated by the first. The studies described here employ activation with an enzyme conjugated to the selective delivery component. CPG2 an enzyme which has no known equivalent activity acting on its specific substrate in a tumour bearing host (Sherwood *et al.*, 1985), has been conjugated to $F(ab')_2$ fragment of a monoclonal antibody W14 (W14-$F(ab')_2$), which is directed at β-hCG, with retention of the specific activity of both enzyme and antibody (Searle *et al.*, 1984). The conjugate localises in CC3, an hCG producing human xenograft choriocarcinoma in nude mice (Melton *et al.*, 1986). It was therefore proposed that activation of prodrugs at CC3 tumour sites could be achieved by prior localisation of the antibody–enzyme conjugate.

Correspondence: K.D. Bagshawe
Received: 6 September 1988.

### Materials and methods

The prodrug para-*N*-bis (2-chloroethyl) aminobenzoyl glutamic acid and its benzoic acid mustard derivative, para-*N*-bis (2-chloroethyl) aminobenzoic acid were synthesised by a modification of the method of Fu (1962). Cleavage of the glutamic acid moiety was performed with CPG2 conjugated to $F(ab')_2$ fragments of W14 anti-βhCG mouse monoclonal antibody. Conversion of prodrug to drug was monitored by change in absorbance and the *Km* determined from the Michaelis–Menten plot. Chemical $t_{1/2}$ of prodrug and drug were determined by addition to sodium perchlorate solution (0.1 M, 10 ml) to give a final concentration of $5 \times 10^{-3}$ M. The reaction was followed to completion by titration of the released acid with NaOH (0.01 M) to a constant pH of 7.4 at 37°C.

CPG2 from *Pseudomonas sp.* (Sherwood *et al.*, 1985) strain RS16 which was cloned and produced in *Escherichia coli* (Minton *et al.*, 1983) catalyses the hydrolytic cleavage of reduced and non-reduced folates to peteroates and L-glutamate. W14-$F(ab')_2$ was conjugated to CPG2 using the heterobifunctional N-maleimidobenzoyl succinimide ester (Searle *et al.*, 1986).

To demonstrate the conversion of prodrug to drug two groups of 4 Nu/Nu mice with CC3 human choriocarcinoma xenografts (Searle *et al.*, 1981) were used. The test group received 30 U of conjugate intravenously followed 24 h later by $16\,mg\,kg^{-1}$ prodrug by the same route; controls received saline and prodrug. 10 μl of blood were diluted 1:10 in saline (0.9%) centrifuged and the supernatant applied to a pre-

**Figure 1** The prodrug *para*-*N*-bis (2-chloroethyl) aminobenzoyl glutamic acid and its benzoic acid mustard derivative, *para*-*N*-bis (2-chloroethyl) aminobenzoic acid formed with cleavage of glutamic acid moiety by carboxypeptidase $G_2$.

treated C18 sample preparation cartridge. The compounds eluted with methanol were dried *in vacuo* and the residue resuspended in 35% acetonitrile: 1% acetic acid. 60–90 $\mu$l was injected into a C18 micro Bondapak (5 $\mu$m) column run in the same solvent at 1 ml min$^{-1}$ and the compounds detected at 305 nm. Retention times were 5 min for the prodrug and 15 min for the drug.

Mean prodrug and drug concentrations were measured in normal tissues and CC3 xenografts. Prodrug was administered intraperitoneally 48 h after intravenous injection of W14-F(ab')$_2$: CPG2 (3 units enzyme activity). The tissues were excised 0.5 h later and frozen at −70° till analysed.

Cytotoxicity of prodrug and drug were tested against JAR human choriocarcinoma cells ($5 \times 10^4$ ml$^{-1}$ grown in DMEM (16) and treated with prodrug or drug in the concentration 5–800 $\mu$M three times at 24-h intervals. CPG2 (6 U ml$^{-1}$ final concentration) was added to equivalent cultures with each dose of prodrug to achieve active drug *in situ*. Cell viability was determined by haemocytometry 24 h after the last treatment.

The effect of antibody enzyme conjugate and prodrug on the growth of CC3 xenografts was compared with saline controls and other controls receiving standard chemotherapeutic agents. The tumours were just palpable on day 1 and all mice had serum HCG >40 IU l$^{-1}$. Tumours were measured twice weekly in 3 diameters and recorded as volume ($\Pi d^1.d^2.d^3/6$) relative to that on day 1. One group of 4 mice received W14 F(ab')$_2$ intravenously (50 U enzyme/mouse) and 56, 72 and 80 h later were given 5 mg prodrug in DMSO/PBS (1:5) intravenously. A second group received 10 mg of prodrug 72, 88 and 96 h later. These treatments were not repeated.

Further groups of 4 mice received intravenously, methotrexate 5 mg kg$^{-1}$, or hydroxyurea 50 mg kg$^{-1}$, or cytosine arabinoside 20 mg kg$^{-1}$, at 0, 16 and 24 h. The 3-dose treatment was repeated weekly until tumour growth required sacrifice. (The mice receiving cytosine arabinoside received 3 doses only).

Other groups of 4 mice in similar condition, received saline in lieu of conjugate and prodrug; or conjugate alone (50 units enzyme); or drug (2.5 mg/mouse × 3 which was maximum tolerated dose); or prodrug alone (10 mg/mouse × 3, maximum tolerated dose 22.5 mg × 3).

### Results

The prodrug *para*-N-bis (2-chloroethyl) aminobenzoyl glutamic acid and its derivative *para*-N-bis (2-chloroethyl) aminobenzoic acid are shown in Figure 1. The extinction coefficient at pH 7.3 and 320 nm was found to be 9,208 L mol$^{-1}$ cm$^{-1}$ for prodrug and 2,062 L mol$^{-1}$ cm$^{-1}$ for drug. CPG2 whether free or conjugated to an antibody cleaves the glutamic acid moiety of the prodrug with high affinity ($K_m = 4.9$ $\mu$M) leaving an active benzoic acid mustard.

**Figure 2** Conversion of prodrug to drug in serum of two groups of 4 Nu/Nu mice with CC3 human choriocarcinoma xenografts receiving antibody enzyme conjugate or saline controls. The test group received 30 units of conjugate intravenously followed 24 h later by 16 mg kg$^{-1}$ prodrug by the same route (● prodrug, ■ drug); controls received saline and prodrug (○ prodrug, △ drug).

The chemical half lives of the prodrug and drug are 16.5 h and 5.3 h respectively.

Evidence of conversion of prodrug to drug *in vivo* was obtained from blood samples taken from xenografted nude mice and the ratio of prodrug to drug when prodrug was given 24 h after administration of W14-F(ab')$_2$:CPG2 conjugate is shown in Figure 2. It is evident that in the presence of conjugate, conversion of prodrug to drug readily occurred with little residual prodrug detectable in plasma. In the absence of conjugate, low levels of drug were detected but only 3 h after injection of the prodrug. The distribution of prodrug and drug in tissues in a similar experiment is shown in Table I. At 0.5 h after prodrug administration prodrug was detectable at all sites except in tumour, a finding consistent with more complete conversion of prodrug to drug at that site than in other tissues.

*In vitro* evidence of cytotoxicity of prodrug and drug on JAR cells is shown in Figure 3. $ID_{50}$ for drug was 20 $\mu$M, whereas prodrug alone at 800 $\mu$M concentration gave only 17% inhibition of control. In the presence of CPG2, prodrug gave an $ID_{50}$ of 8 $\mu$M. No loss of viability was detected in the presence of enzyme alone.

*In vivo* evidence of antitumour activity is shown in Figure 4. Groups receiving conjugate and prodrug showed marked suppression of tumour growth (Figure 4a). Prodrug alone produced slight growth delay. Tumours in mice receiving

**Table I** Mean prodrug and drug concentrations ($\mu g\,g^{-1}$) in normal tissues and CC3 xenografts in Nu/Nu mice. Prodrug was administered intraperitoneally 48 h after intravenous injection of W14-F(ab')$_2$:CPG2 (30 U enzyme activity). The tissues were excised 0.5 h later

| *Tissue* | *Prodrug* | *Drug* |
|---|---|---|
| Tumour | <0.05 | 0.86 |
| Liver | 22.0 | 7.5 |
| Kidney | 2.9 | 2.8 |
| Lung | 0.20 | 2.5 |
| Gut | 12.3 | 1.3 |
| Spleen | 0.51 | 1.5 |
| Brain | 0.16 | 0.65 |

**Figure 3** Cytotoxicity of prodrug and drug against JAR human choriocarcinoma cell line. JAR cells ($5 \times 10^4$/ml) were grown in DMEM and treated with prodrug (●) or drug (○) in the concentration range 5–800 $\mu$M three times at 24 h intervals. CPG2 ($6\,U\,ml^{-1}$ final concentration) was added to equivalent cultures with each dose of prodrug (▲) to achieve active drug *in situ*.

conventional cytotoxic agents, given in full dosage three times in a 24-h period, at weekly intervals, showed either growth at a similar rate to saline controls or accelerated growth. Drug alone given at maximum tolerated dose showed no inhibition of tumour growth (Figure 4b).

### Discussion

The principle of converting relatively inert prodrugs into active cytotoxic agents by enzymes is well established but this has not been achieved specifically at cancer sites, because human cancers have not been found to exhibit intrinsic enzymic activity sufficiently distinctive from normal tissues. The ability of a localised non-mammalian enzyme to generate an effective concentration of cytotoxic molecules from an appropriate prodrug in a tumour is indicated by the marked inhibition of growth achieved here in contrast to the ineffectiveness of the drug and of conventional cytotoxic agents given intravenously.

The tumour and prodrug used here constitute a rigorous test for this approach. The target antigen is freely secreted by differentiated syncytiotrophoblastic cells but not by the clonogenic cytotrophoblast cells (Midgley & Pierce, 1962) and the antibody–enzyme conjugate encounters a high concentration of antigen in body fluids. The CC3 tumour as shown here is resistant to a wide range of conventional cytotoxic agents. Although the prodrug biological $t_{1/2}$ (biphasic = $\alpha$ 0.32 h, $\beta$ = 1.8 h) was satisfactory the active drug has an undesirably long half-life (biphasic $\alpha$ = 0.5 h, and $\beta$ = 1.7 h) allowing it time to diffuse away from activation sites and to exert toxic effects. Despite these limitations it is encouraging that marked inhibition of growth of this tumour was observed with a single course of treatment.

An antibody–enzyme conjugate is unlikely to have better penetration or more uniform distribution within a tumour than antibody itself, but activation of a prodrug at tumour sites by the enzyme has several potential advantages. The prodrug can be relatively inert and have a long biological half-life. It is desirable that the drug released from the prodrug should be small, readily diffusible and able to enter cells, or be toxic at membrane sites whether or not they express target antigen. The limitations of poor penetration

**Figure 4** Growth curves for CC3 choriocarcinoma xenografts in Nu/Nu mice following injection with various agents.

(a) Two groups of 4 mice received W14 F(ab')$_2$:CPG2 intravenously (50 units enzyme/mouse) and 56, 72 and 80 h later; one group (○) were injected intravenously with 5 mg prodrug in DMSO/PBS (1:5); the other group (●) received 10 mg of prodrug 72, 88 and 96 h later. These treatments were not repeated.

Further groups of 4 mice received standard chemotherapeutic iv: methotrexate $5\,mg\,kg^{-1}$ (■); hydroxyurea $50\,mg\,kg^{-1}$ (▲); actinomycin D $7.5\,\mu g\,kg^{-1}$ (X); cyclophosphamide 20 mg kg– (□); cytosine arabinoside $20\,mg\,kg^{-1}$ (△) at 0, 16 and 24 h. The 3-dose treatment was repeated weekly until tumour growth required sacrifice, except for mice receiving cytosine arabinoside which received 3 doses only.

(b) Mice in similar condition to those in 4(a) received saline *in lieu* of conjugate and prodrug (△); or conjugate alone (50 units enzyme) (▲); drug (2.5 mg mouse$^{-1}$ × 3 which was maximum tolerated dose) (●); or prodrug alone (10 mg mouse$^{-1}$ × 3, maximum tolerated dose 22.5 mg × 3) (○).

by antibody and heterogeneity in distribution of target antigen can therefore be overcome. Normal tissues can be protected by the favourable distribution of the activating enzyme and by ensuring that the released active drug has a short half-life. In this way it should be possible to generate prolonged high concentrations of cytotoxic drugs in antibody binding tumours without inducing serious normal tissue toxicity. Drug resistance is potentially avoidable by mass drug action and if necessary, by multiple prodrugs.

The first drug we have tested in this system falls short of the ideal mainly with respect to its prolonged half-life. It is evident however that various classes of prodrug can be synthesised for activation by a range of matching enzymes of non-human origin. Translation of this approach to the clinic is likely to encounter logistical problems in the production of antibody–enzyme conjugate on a large enough scale whether by chemical conjugation or genetic engineering. These problems now need to be addressed.

We thank the Cancer Research Campaign for grant support, Dr M. Jarman for advice, the Institute of Cancer Research for facilities and Joan and Robert Boden for skilled technical assistance.

**References**

BAGSHAWE, K.D. (1987). Antibody directed enzymes revive anti-cancer prodrugs concept. *Br. J. Cancer*, **56**, 531–532.

BEGENT, R.H.J. (1986). Antibody localisation in tumours: The theoretical basis and clinical applications in imaging. *Ann. Acad. Med.*, **15**, 561–566.

CHAPMAN, C.E., FAIRWEATHER, D.S., KEELING, A.A. & 3 others (1986). Clinical evaluation of anti-alpha-fetoprotein radioimmuno-detection. *Brit. J. Radiol.*, **59**, 1175–1178.

CHARPIN, C., BHAN, A.K., ZURAWSKI, V.R. & SCULLY, R.E. (1982). Carcinoembryonic antigen (CEA) and carbohydrate determinant 19-9 (CA19-9) localisation in 121 primary and metastatic ovarian tumours: An immunohistochemical study with the use of monoclonal antibodies. *Int. J. Gynecol. Pathol.*, **1**, 231–245.

FRANKEL, A.E. (1985). Antibody–toxin hybrids: A clinical review of their use. *J. Biolog. Response Modifiers*, **4**, 437–446.

FU, S.-CJ. (1962). Para-[*N*-bis-(2-chloroethyl)]-aminobenzoylglutamic acid. *J. Med. Pharm. Chem.*, **5**, 33–41.

KANELLOS, J., PEITERSZ, G.A. & McKENZIE, I.F.C. (1985). Studies of methotrexate–monoclonal antibody conjugates for immunotherapy. *J. Nat. Cancer Inst.*, **75**, 319–329.

KEMSHEAD, J.T., JONES, D.H., LASHFORD, L. & 4 others (1986). 131-I coupled to monoclonal antibodies as therapeutic agents for neuroectodermally derived tumors: Fact or Fiction. *Cancer Drug Delivery*, **3**, 25–43.

LEDERMANN, J.A., BEGENT, R.H.J. & BAGSHAWE, K.D. (1988). Cyclosporin A prevents the anti-murine antibody response to a monoclonal anti-tumour antibody in rabbits. *Br. J. Cancer* (in press).

LEDERMANN, J.A., BEGENT, R.H.J., BAGSHAWE, K.D. & 5 others (1988). Repeated antitumour antibody therapy in man with suppression of the host response by Cyclosporin A. *Br. J. Cancer* (in press).

LEWIS, J.C.M., BAGSHAWE, K.D. & KEEP, P.A. (1982). The distribution of parenterally administered antibody to CEA in colorectal xenografts. *Oncodevelopmental Biol Med.*, **3**, 161–168.

MELTON, R., SEARLE, F., BIER, C. & 5 others (1988). Antibody:carboxypeptidase G2 conjugates as potential tumour imaging agents. *Proc. NATO Workshop on Monoclonal Antibodies for Imaging and Therapy*. Castel Vecchio, Cascoli, 377–380.

MIDGLEY, A.R. & PIERCE, G.B. (1982). Immunohistochemical localisation of human chorionic gonadotropin. *J. Exp. Med.*, **115**, 289–294.

MINTON, N.P., ATKINSON, T. & SHERWOOD, R.F. (1983). Molecular cloning of the Pseudomonas carboxypeptidase G2 gene and its expression in *Escherichia coli* and *Psudomonas putida*. *J. Bacteriol.*, **156**, 1222–1227.

OHUCHI, N., SIMPSON, J.F., COLCHER, D. & SCHLOM, J. (1987). Complementation of anti-CEA and anti-tag-72 monoclonal antibodies in reactivity to human gastric adenocarcinomas. *Int. J. Cancer*, **40**, 726–733.

PRIMUS, F.J., KUHNS, W.J. & GOLDENBERG, D.M. (1983). Immunological heterogeneity of carcinoembryonic antigen determinants in colonic tumors with monoclonal antibodies. *Cancer Res.*, **43**, 693–701.

ROGERS, G.T., HARWOOD, P.J., PEDLEY, R.B. & 3 others (1986). Dynamics of monoclonal antibody distribution and prolonged tumour localisation in nude mice bearing a human CEA-producing colon carcinoma xenograft. *Tumour Biol.*, **6**, 453–463.

SEARLE, F., PARTRIDGE, C.S., KARDANA, A. & 4 others (1984). Preparation and properties of a mouse monoclonal antibody (W14A) to human chorionic gonadotrophin. *Int. J. Cancer*, **33**, 429–434.

SEARLE, F., BIER, C., BUCKLEY, R.G. & 4 others (1986). The potential of carboxypeptidase $G_2$ antibody conjugates as anti-tumour agents. 1. Preparation of antihuman chorionic gonadotrophin carboxypeptidase $G_2$ and cytotoxicity of the conjugates against JAR choriocarcinoma cells *in vitro*. *Br. J. Cancer*, **53**, 377–384.

SEARLE, F., BODEN, J., LEWIS, J.C.M. & BAGSHAWE, K.D. (1981). A human choriocarcinoma xenograft in nude mice: A model for the study of antibody localisation. *Br. J. Cancer*, **44**, 137–144.

SHERWOOD, R.F., MELTON, R.G., ALWAN, S.M. & HUGHES, P. (1985). Purification and properties of carboxypeptidase $G_2$ from *Psuedomonas sp.* strain RS-16. Use of a novel triazine dye affinity method. *Europ. J. Biochem.*, **148**, 447–453.

SHIN, H.S., PASTERNACK, G.R., ECONOMOU, J.S. & 2 others (1976). Immunotherapy of cancer with antibody. *Science*, **194**, 327–329.

VAN NAGELL, J.R. JR., KIM, E., CASPER, S. & 4 others (1980). Radioimmunodetection of primary and metastatic ovarian cancer using radiolabelled antibodies to carcinoembryonic antigen. *Cancer Res.*, **40**, 502–506.